# Spermidine Retarded the Senescence of Multipotent Mesenchymal Stromal Cells *In Vitro* and *In Vivo* through SIRT3-Mediated Antioxidation

**DOI:** 10.1155/2023/9672658

**Published:** 2023-05-17

**Authors:** Hua Huang, Wen Zhang, Junjie Su, Bisheng Zhou, Qingjiang Han

**Affiliations:** ^1^Department of Urology, The First Affiliated Hospital and College of Clinical Medicine of Henan University of Science and Technology, Luoyang 471003, China; ^2^The Center of Reproductive Medicine, The First Affiliated Hospital and College of Clinical Medicine of Henan University of Science and Technology, Luoyang 471003, China; ^3^Department of General Medicine, The First Affiliated Hospital and College of Clinical Medicine of Henan University of Science and Technology, Luoyang 471003, China

## Abstract

Multipotent mesenchymal stromal cells (MSCs) expand *in vitro* and undergo replicative senescence, thereby restricting their clinical utilization. Thus, an effective strategy is required to impede MSC senescence. Since spermidine (SPD) supplementation can prolong the lifespan of yeast by inhibiting oxidative stress, spermidine is a potential option for delaying MSC senescence. In this study, to test our hypothesis, we first isolated primary human umbilical cord mesenchymal stem cells (hUCMSCs). Subsequently, the appropriate SPD dose was administered during continuous cell cultivation. Next, we evaluated the antisenescence effects by SA-*β*-gal staining, Ki67 expression, reactive oxygen species (ROS) levels, adipogenic or osteogenic ability, senescence-associated markers, and DNA damage markers. The results revealed that early SPD intervention significantly delays the replicative senescence of hUCMSCs and constrains premature H_2_O_2_-induced senescence. Additionally, by silencing SIRT3, the SPD-mediated antisenescence effects disappear, further demonstrating that SIRT3 is necessary for SPD to exert its antisenescence effects on hUCMSCs. Besides, the findings of this study also suggest that SPD *in vivo* protects MSCs against oxidative stress and delays cell senescence. Thus, MSCs maintain the ability to proliferate and differentiate efficiently *in vitro* and *in vivo*, which reflects the potential clinical utilization of MSCs in the future.

## 1. Introduction

Multipotent mesenchymal stromal cells (MSCs), particularly those derived from the human umbilical cord tissue [1–4], possess osteogenic, chondrogenic, and adipogenic abilities. However, the dysfunction or numerical reduction of MSCs may lead to certain pathological changes and clinical symptoms. For premature aging disorders such as Hutchinson-Gilford progeria syndrome or Werner syndrome, accelerated attrition of the MSC pool has been detected [5]. Conversely, the extension of lifespan has been observed in mice by the transplantation of young MSCs [6]. Therefore, MSCs can potentially be applied in the field of inflammatory diseases and regenerative medicine [7–10]. Firstly, before clinical application, MSCs must be expanded *in vitro* to obtain sufficient cells. [11–14]. However, as MSCs expand, they undergo replicative senescence, including decreased proliferation and differentiation, which greatly restricts their clinical utilization potential [15–17]. These negative effects are generally due to intracellular factors such as high reactive oxygen species (ROS), oxidative stress, telomere shortening, DNA damage, and extracellular factors involving the stem cell niche [18]. As a result, an effective strategy is essential for delaying MSC senescence.

Certain substances such as antioxidants, vitamins, hormones, and plant extracts exhibit antiaging effects [19]. However, they exert a weak effect on stem cell senescence. Spermidine (SPD), a natural small molecule compound that belongs to polyamines, mainly exists in the semen. It also exists in our daily diet and is essential for the proper function of numerous metabolic processes [20–23]. The external supply of natural spermidine extends lifespan by inhibiting oxidative stress in model organisms including nematodes, yeast, flies, and mouse flies [24–27]. Moreover, some evidence suggests that spermidine also delays neurodegeneration in nonmammalian model organisms or mouse models [28]. However, there are no conclusive reports on whether spermidine delays the cellular senescence of stem cells. Based on this, we speculate that SPD may delay the senescence of MSCs *in vitro* or *in vivo*. Sirtuins are a widely known type of the NAD+-dependent protein deacetylase, and the seven sirtuins (SIRT1, SIRT2, SIRT3, SIRT4, SIRT5, SIRT6, and SIRT7) that have been identified in humans play a vital role in antiaging effects [29]. Among them, SIRT3 is localized in the mitochondrial matrix, which is a key regulator of mitochondrial function that decreases mitochondrial ROS and reduces oxidative stress [30, 31]. Studies have shown that SIRT3 ameliorates lung senescence by enhancing the antioxidant defense mechanism [32]. Moreover, SIRT3 deficiency shortens the lifespan of myocardial mitochondria and impairs their function [33]. However, the relationship between the role of SIRT3 during the aging of stem cells and the effect of spermidine on SIRT3 remains unclear. In this study, we aim to demonstrate that spermidine restricts the cellular senescence of stem cells and reveal the role of SIRT3 in antiaging effects mediated by spermidine.

To verify our hypothesis, we isolated the primary human umbilical cord mesenchymal stem cells (hUCMSCs) and expanded them continuously *in vitro* both with and without SPD. Additionally, a long-term SPD diet as a treatment factor was applied to explore whether SPD could also slow down the MSC aging process *in vivo*. The results of this study demonstrate that early SPD intervention significantly delays the replicative senescence of hUCMSCs and inhibits H_2_O_2_-induced premature senescence. Furthermore, SIRT3 is required to achieve SPD-mediated antisenescence effects. Besides, this study also reveals that SPD administration *in vivo* protects MSCs against oxidative stress and delayed cell senescence.

## 2. Materials and Methods

### 2.1. Cell Isolation and Culture

Primary hUCMSCs were isolated and cultured according to the procedures of a previous study [34]. In brief, after the informed consent was obtained from women who underwent cesarean section, the collection and subsequent use of the umbilical cord were approved by the Institutional Ethical Review Committee of the First Affiliated Hospital of Henan University of Science and Technology. Aseptic umbilical cord tissue was collected after cesarean delivery by a healthy child-bearing-age mother, according to the guidelines of the World Medical Association Declaration of Helsinki. A length of around 3 cm of Wharton's jelly was cut into 1-2 mm^3^ pieces and then plated in 10 cm dishes (Corning, Acton, MA, USA) containing DMEM/low-glucose medium (Gibco, USA) with 10% fetal bovine serum (FBS, HyClone) and 1% penicillin-streptomycin. It was then cultivated at 37°C in a 5% CO_2_ atmosphere in a humidified incubator. Cells were subcultured at a density of 5,000 cells/cm^2^ until they had grown to 70% confluence, and the medium was changed every 3 days. Population doubling levels (PDL) were counted: 1PDL = Log_10_ (*N*/*N*_0_) × 3.33 (*N* is the number of cells at the end of a passage, and *N*_0_ is the number of cells that were seeded at the beginning of a passage). The cell type was identified by flow cytometry (BD Biosciences) using a Human MSC Analysis Kit (BD Pharmingen, San Diego, CA, USA), per our previous study [35].

### 2.2. Cell Viability Assay

Cell viability was tested with the Cell Counting Kit-8 assay (CCK-8, Solarbio, China) according to the guidelines of the manufacturer. hUCMSCs were precultured in a 96-well plastic microtiter containing 10% FBS solution for 24 h and subsequently treated with different concentrations of SPD (Sigma, USA) for an additional 24 h. Cells were further treated with 10 *μ*l CCK-8 solution for 4 h, and the absorbance was read using a microplate reader at a 450 nm wavelength.

### 2.3. SPD Treatment and Cell Senescence *In Vitro*

Early SPD intervention was performed by supplementing the cell medium with different concentrations of SPD, as the figures indicate. The replicative senescence of hUMSCs was determined through a continuous subculture, as previously described [36]. The premature senescence of hUCMSCs was established by adding 100 *μ*M H_2_O_2_ to the medium after SPD treatment, and the medium was refreshed every 48 h with medium containing 10 *μ*M SPD.

### 2.4. SA-*β*-Galactosidase Staining

The senescent status of hUMSCs and rat adipose tissue-derived-mesenchymal stem cells (rADMSCs) was measured by in situ staining using the senescence-associated *β*-galactosidase (SA-*β*-gal) staining kit (Cell Signaling Technology, MA, USA), following the manufacturer's instructions. Briefly, cells grown on 6-well dishes were washed once with 1X PBS, fixed for 15 min with 1x fixative solution, and rinsed twice with 1x PBS. Subsequently, 1 ml *β*-galactosidase staining solution was added to each well and the plate was incubated at 37°C overnight in a CO_2_-free dry incubator. SA-*β*-gal-positive cells appeared blue, and the number of positively stained cells was calculated for every 200 cells in randomly selected fields of vision under light microscopy (Leica DMi8, Germany).

### 2.5. ROS Detection

To measure the levels of intracellular ROS, an H2DCFDA-Cellular ROS Assay Kit (ab113851, Abcam, Cambridge, UK) was used. In brief, hUCMSCs were first plated in 6-well dishes and pretreated with or without 10 *μ*M SPD for 24 h; then, they were treated with 100 *μ*M for an additional 24 h. Subsequently, the cells were washed using serum-free medium and incubated with 10 *μ*M 2′,7′-dihydrodichlorofluorescein diacetate (H2DCFDA) at 37°C for 30 min in the dark. After removing the medium, the cells were washed and then observed under a fluorescence microscope (Leica DMi8, Germany). To quantify the ROS level, the H2DCFDA fluorescence intensity of the cells was detected using flow cytometry. Specifically, 1 × 10^5^ cells were collected in each group and incubated in 10 mM H2DCFDA with serum-free medium. Next, the cells were washed with serum-free medium for 20 min with 5% CO_2_ at 37°C. Finally, ROS levels were detected using fluorescence-activated cell sorting (FACS; BD Biosciences).

### 2.6. Cell Differentiation Assays

The multilineage differentiation ability of hUCMSCs was performed according to previously described methods [37]. Briefly, cells were seeded in a 24-well plate and grown to 70% confluency. Osteogenic and adipogenic differentiation media (STEMCELL Technologies, Canada) were added and refreshed every 3 days. After 2 weeks of cultivation, osteogenic differentiation was evaluated with Alizarin Red S staining (Sigma-Aldrich, USA). Following 3 weeks of culturing, adipogenic differentiations were assessed using Oil Red O staining (Sigma-Aldrich, USA). Alizarin Red S staining and Oil Red O staining were quantified by reading the absorbance at 520 nm and 500 nm, respectively.

### 2.7. Western Blotting

hUCMSCs were collected and lysed in 1x RIPA buffer (Solarbio, China) containing protease inhibitors (Sangon Biotech, China) on ice for 30 minutes. Protein lysate was separated through 10% SDS-PAGE and then transferred to nitrocellulose filter (NC) membranes (Millipore, USA). The membranes were blocked with a blocking solution (5% skim milk in TBST) for 1 h at room temperature and then incubated with primary antibodies, including rabbit anti-human P21, p-P53-ser15, P53, SIRT3, OCT4, SOX2, and GAPDH (1 : 2000, Abcam, Cambridge, UK) and rabbit anti-human ALP, RUNX2, PPAR*γ*, and FABP4 (1 : 1000, Biorbyt, San Francisco, CA, USA) overnight at 4°C. Furthermore, the NC membranes were washed and incubated with goat anti-rabbit antibody (1 : 10000, Abcam) for 1 h at room temperature in the dark. The special bands were visualized using a two-color infrared laser imaging system (LI-COR, Odyssey, USA). Protein expression levels detected by western blotting were quantified using ImageJ software (version 1.52U, NIH, USA).

### 2.8. Immunofluorescence Staining

To detect DNA damage and the proliferative activity of cell *γ*-H2AX, Ki67 immunofluorescence staining was performed. Specifically, cells were fixed with 4% paraformaldehyde and exposed to 0.25% Triton X-100 (Sigma, USA) in PBS solution for 10 min at room temperature. The cells were then blocked with 5% BSA solution for 30 min and incubated with the primary antibody anti-*γ*H2AX or anti-Ki67 (1 : 100, Abcam) overnight at 4°C. After washing the cells with PBS, the cells were further incubated with a fluorescently tagged secondary antibody (1 : 500, Abcam) for 1 h at room temperature in the dark. Subsequently, Hoechst 33342 (20 *μ*g/ml, Yeasen, China) was used to stain the nuclei. Finally, immunofluorescence staining was observed under a fluorescence microscope (Leica DMi8, Germany).

### 2.9. siRNA Silencing

In this study, the small interfering RNA (siRNA) targeted the sequence of the sirtuin-3 gene (SIRT3): sense, 5′-CTCCTCTGTTGCCTTGGTA-3′ for SIRT3-1, 5′-GCGCCTATCAGTACACAAT-3′ for SIRT3-2, 5′-CAGCAAGGUUCUUACUACATT-3′ for SIRT3-3, or scrambled 5′-CAACAAGATGAAGAGCACC-3′ (Genomeditech Co. Ltd., China). The hUCMSCs were transfected with 100 nmol human siSIRT3 or siScrambled using a FuGENE® 6 transfection reagent (Promega, Wisconsin, USA) and optimedium supplemented with 10% FBS overnight, according to the manufacturer's directions. Furthermore, 72 h after siRNA transfection, the cells were harvested and the efficiency of SIRT3 silencing was evaluated according to the expression levels of SIRT3 detected by western blotting.

### 2.10. Animals and Treatment

Middle-aged (18 months) male Sprague-Dawley rats were obtained from the Experimental Animal Center of Henan Province (Zhengzhou, China) and kept under a 12 h light/dark cycle, controlled temperature (23°C), and constant relative humidity of 50%-60%. Food and drinking water were freely accessible. The rats were randomly divided into two groups: the control group (*N* = 40) and the spermidine group (*N* = 40). Rats in the spermidine group were treated with 30 mg/kg/day spermidine (Sigma-Aldrich, St. Louis, MO, S0266) dissolved in drinking water for at least a year. Conversely, the control group rats received water only. All surgical processes and postoperative care were approved by the Ethics Committee at the First Affiliated Hospital of Henan University of Science and Technology (no. 2021-03-B044) and carried out according to Institutional Animal Care and Use Committee guidelines.

### 2.11. Bioavailability of Spermidine

Serum from the control and treated rats was collected by cardiac puncture, while spermidine concentration was detected using an immunoassay (BioCatGmbH, Heidelberg, Germany).

### 2.12. rADMSC Isolation

rADMSCs were isolated in a straightforward process described in a previous study [38]. Briefly, the stromal vascular fraction (SVF) was isolated from the minced subcutaneous adipose tissue of rats and digested with 0.1% type I collagenase solution (Slorbia, Beijing, China) for 1 hour at 37°C. After filtration through 45 *μ*m strainers (Corning, Acton, MA, USA) and centrifugation (1,500 rpm for 15 min at 4°C), the floating fat cells were removed. The pellet was resuspended in *α*MEM (HyClone, USA) containing 10% fetal bovine serum (FBS, HyClone) and 1% penicillin-streptomycin. Cell subcultures were regularly kept at 70% cell confluence, and the medium was renewed every 2-3 days.

### 2.13. Statistical Analysis

Data are presented as a mean ± standard error of the mean [39]. The significance of the difference between groups was analyzed via two-tailed Student's *t*-test or ANOVA using GraphPad Prism 6 software (GraphPad Software, Inc., La Jolla, CA, USA). A value of *p* < 0.05 (^∗^) is considered statistically significant.

## 3. Results

### 3.1. Effect of SPD on Cell Viability

**Figure 1:**
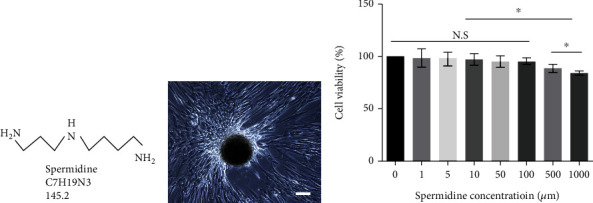
Primary cell separation and cell viability test. (a) Structural and molecular formulas of small molecule compound spermidine. (b) Primary hUCMSCs harvested by the tissue block method and identified by our existing methods [35]. (c) Cell viability was tested with CCK-8, different concentrations of spermidine were added to the medium, and absorbance was measured using a microplate reader after 12 h. Cells were seeded in 96-well plates at a concentration of 10^4^ cells per well. All quantitative data were obtained from three independent experiments and presented as mean ± SEM; ^∗^*p* < 0.05 indicates a significant difference between the specified groups. N.S.: not significant; scale = 100 *μ*m.

Spermidine, a naturally occurring small molecule compound with a unique role in physiological function, is attracting increasing attention from numerous researchers (Figure 1(a)). In this study, to evaluate the effect of SPD on the cell viability of hUCMSCs *in vitro*, hUCMSCs were first isolated from a sterile umbilical cord tissue using the tissue block stick method. We observed that spindle-like cells migrated from the tissue fragment after 10 days (Figure 1(b)). These isolated cells were identified by surface markers CD90, CD105, and CD73, according to our previously reported method, with a purity exceeding 90% [35]. Next, the toxicity of SPD on hUCMSCs was assessed with CCK-8. The results revealed that SPD did not affect the cell viability of hUCMSCs at low doses (*p* > 0.05), while cell viability was slightly reduced when the dose was higher than 100 *μ*M (*p* < 0.05; Figure 1(c)). Therefore, the optimal dose for the following experiments is between 0 *μ*M and 100 *μ*M.

### 3.2. SPD Intervention Delays hUCMSC Replicative Senescence

**Figure 2:**
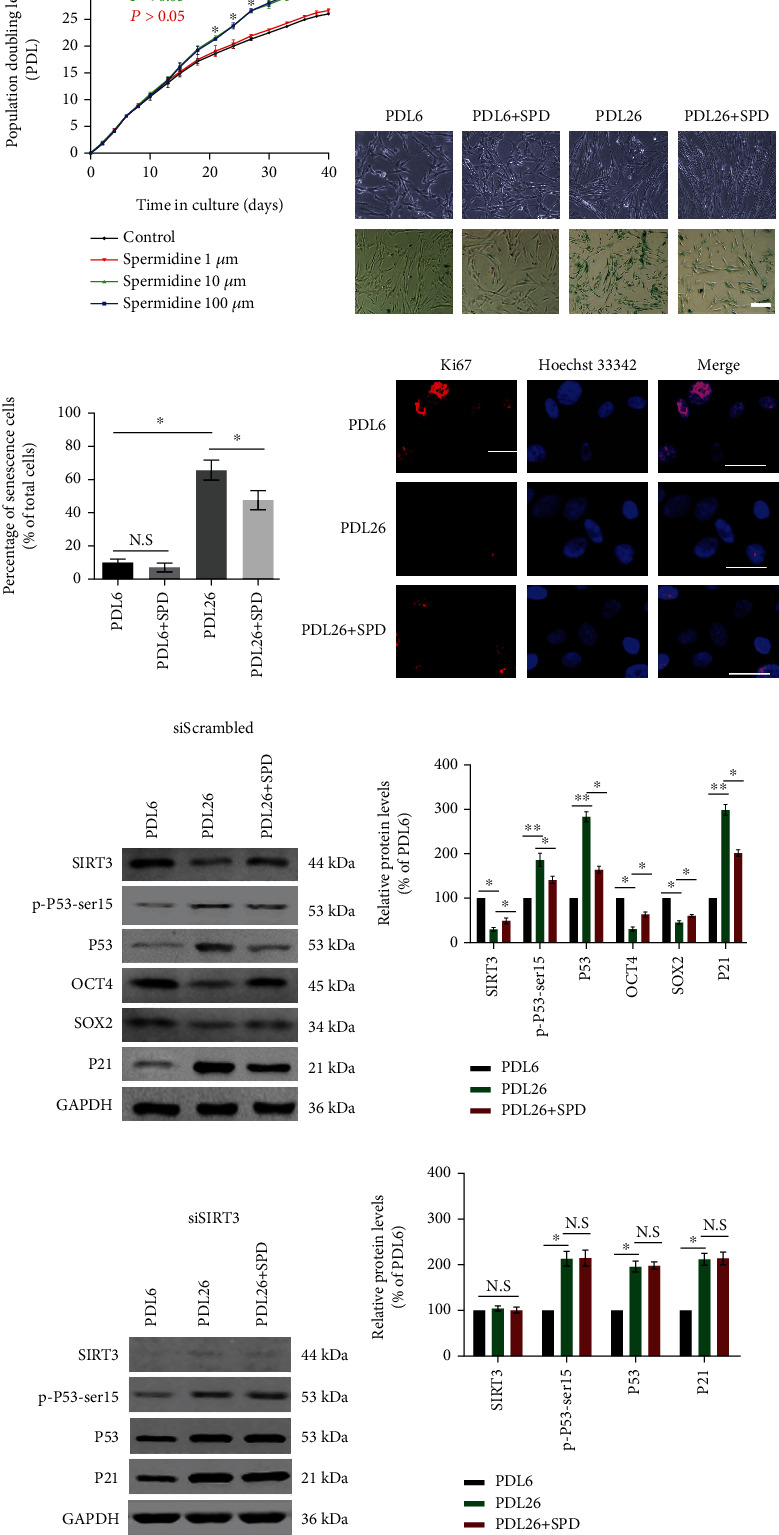
SPD intervention delayed hUCMSC replicative senescence. (a) The replicative potential of hUCMSCs cocultivated with 0 (control), 1, 10, or 100 *μ*M SPD, respectively, is expressed as the population doubling time (population doubling level vs. time). (b) A representative micrograph including the SA-*β*-gal staining of hUCMSCs from earlier passages (PDL6), later passages (PDL26), and later passage cells supplemented with 10 *μ*M SPD during subculture (PDL26+SPD). (c) The percentage of senescent cells stained in blue in each group was assessed by quantitative analysis. (d) Immunofluorescence staining of Ki67 foci appeared red, while blue indicated nuclei stained with Hoechst 33342; scale = 50 *μ*m. (e) Representative western blotting results for the protein expression of SIRT3, p-P53-ser15, P53, OCT4, SOX2, or P21 in different groups. (f) Protein expression levels detected by western blotting were quantified using ImageJ software and normalized to PDL6. (g) Representative western blotting results for the protein expression of SIRT3, p-P53-ser15, P53, or P21 in different groups when SIRT3 was silenced. (h) Protein expression levels detected by western blotting were quantified and normalized to PDL6. Approximately 10^5^ cells per well were seeded in 6-well plates. All quantitative data were obtained from three independent experiments and presented as mean ± SEM; ^∗^*p* < 0.05 and ^∗∗^*p* < 0.01 signify a significant difference between the indicated groups. N.S.: not significant; scale = 100 *μ*m.

Firstly, the proliferation rate of hUCMSCs was assessed according to the population doubling level (PDL). It indicated that the PDL was significantly higher in the medium supplemented with 10 *μ*M or 100 *μ*M SPD than in the medium with 0 *μ*M or 1 *μ*M SPD (*p* < 0.05 on day 21). Also, there was no significant difference between 10 *μ*M and 100 *μ*M SPD (Figure 2(a)). Additionally, the proliferative activity of cells was evaluated through the expression levels of Ki67. As predicted, the expression levels of Ki67 in the PDL26 group without SPD were negligible, while in PDL26 with SPD treatment, there was medium expression (Figure 2(d)). Evaluation of cell senescence was performed through SA-*β*-gal staining, and the expression levels of senescence-associated factors (P53 and P21) and the longevity-related factor (SIRT3) were detected by western blotting at the corresponding PDL. The proportion of positive SA-*β*-gal-stained cells was substantially higher in the later-passage cells (66.3% ± 5.2% for PDL26) than in the earlier-passage cells (10.6% ± 2.3% for PDL6; *p* < 0.05). However, the proportion fell significantly during *in vitro* subculture supplemented with 10 *μ*M SPD (46.4% ± 4.3% for PDL + SPD; Figures 2(b) and 2(c)). Interestingly, the protein levels of SIRT3 dropped during cell senescence and partially recovered when hUCMSCs were cocultured with SPD (Figures 2(e) and 2(f)). However, the expression of senescence-associated protein P21 or P53 increased during long-term subculture without SPD but decreased under early SPD intervention (Figures 2(e) and 2(f)). Moreover, the levels of phosphorated P53 (p-P53-ser15), an active form of P53, presented a similar trend to P53 and P21 (Figures 2(e) and 2(f)). Additionally, the expression levels of stemness-associated markers OCT4 and SOX2 decreased considerably during cell senescence and partially recovered after early SPD intervention (Figures 2(e) and 2(f)). However, the levels of senescence-associated proteins p-P53-ser15, P53, and P21 increased significantly during long-term subculture when the SIRT3 gene was silenced. Moreover, early intervention of SPD did not inhibit the expression of senescence-associated genes p-P53-ser15, P53, and P21 (Figures 2(g) and 2(h)). These results suggested that long-term subculture led to hUCMSC replicative senescence and early SPD intervention significantly delayed the process. Mechanically, SPD delayed the replicative senescence of hUCMSCs by regulating SIRT3.

### 3.3. SPD Intervention Facilitates Multilineage Differentiation Potential in Later-Passage hUCMSCs

**Figure 3:**
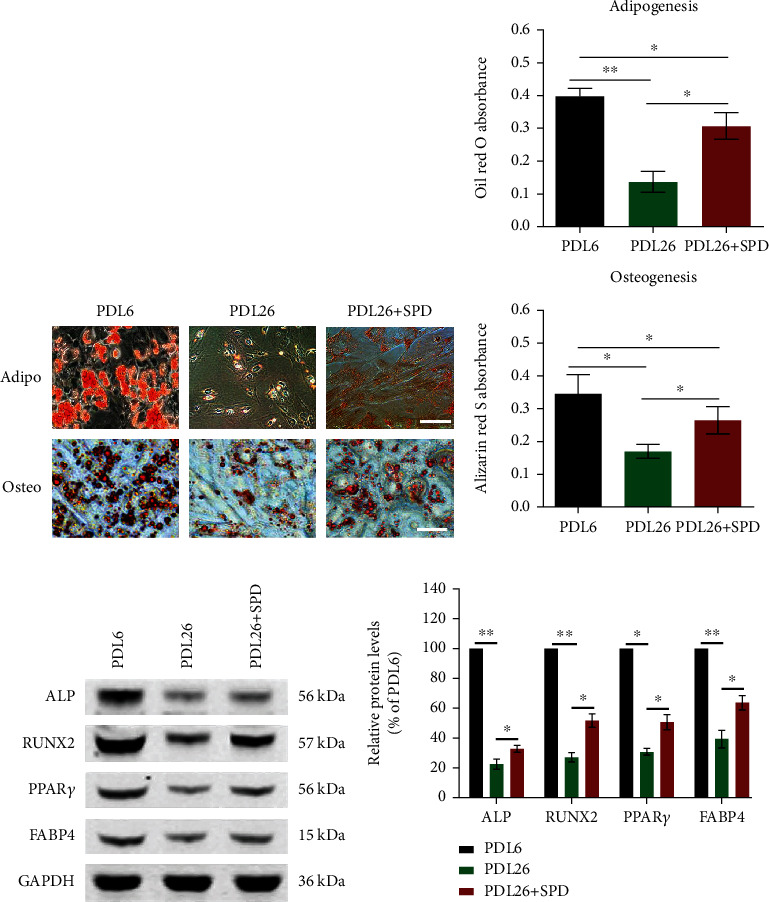
SPD intervention contributed to adipogenic and osteogenic differentiation in later-passage hUCMSCs. (a) Osteogenic and adipogenic differentiation was, respectively, observed 14 days and 21 days after treatment with the corresponding induced medium. Approximately 10^5^ cells per well were seeded in 6-well plates. Adipocytes were stained with Oil Red O, and osteoblasts were stained with Alizarin Red S. (b) The absorbance value was detected at 500 nm (Oil Red O) or 520 nm (Alizarin Red S) and then quantitatively analyzed between different groups. (c) Representative western blotting results indicated the protein expression levels of FABP4, PPAR*γ*, ALP, or RUNX2 in various groups. (d) Protein expression levels detected by western blotting were quantified using ImageJ software and normalized to PDL6. All quantitative data were obtained from three independent experiments and presented as mean ± SEM; ^∗^*p* < 0.05 and ^∗∗^*p* < 0.01 imply a significant difference between the indicated groups, scale = 100 *μ*m.

It is widely recognized that stem cells can self-renew and replicate, while they also possess the potential for multidirectional differentiation. In this study, to evaluate the multilineage differentiation ability of later-passage hUCMSCs, these isolated primary cells underwent a long-term subculture (PDL26). Osteogenic and adipogenic differentiation was observed by Oil Red O and Alizarin Red S staining, respectively, 14 days and 21 days after treatment with the corresponding induced medium. Our findings revealed that the later-passage hUCMSCs (PDL26) significantly declined in osteogenic and adipogenic abilities compared with the earlier-passage cells (PDL6), while early SPD intervention (10 *μ*M) significantly facilitated differentiation toward both osteoblasts and adipocytes (Figures 3(a) and 3(b)). Furthermore, western blotting was performed to detect the levels of adipogenesis (FABP4 and PPAR*γ*) or osteogenesis-associated factors (ALP and RUNX2). Similarly, results showed that early SPD supplementation enabled the maintenance of adipogenic and osteogenic differentiation (Figures 3(c) and 3(d)).

### 3.4. SPD Protects hUCMSCs from H_2_O_2_-Induced Premature Senescence

**Figure 4:**
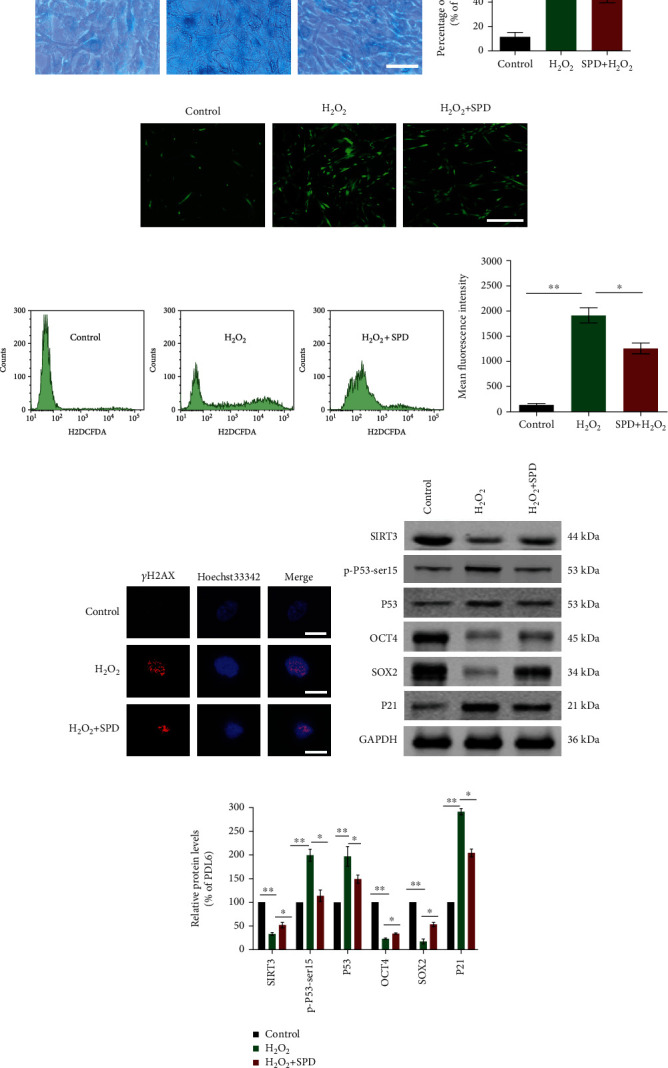
SPD blocked H_2_O_2_-induced hUCMSCs senescence. (a) A representative image showing SA-*β*-gal staining in hUCMSCs at PDL6 (control), cells treated with 100 *μ*M H_2_O_2_, and cells precultivated with 10 *μ*M SPD for 24 h before H_2_O_2_ treatment (H_2_O_2_+SPD); scale = 100 *μ*m. (b) The percentage of senescent cells stained in blue in each group was assessed by quantitative analysis. (c) A representative image showing intracellular ROS levels by molecular probe H2DCFDA staining in hUCMSCs with indicated treatment; scale = 100 *μ*m. (d, e) Quantification of intracellular ROS levels was performed using fluorescence-activated cell sorting (FACS) analysis of stained cells to obtain the mean fluorescence intensity. (f) Immunofluorescence staining of *γ*-H2AX foci appearing red indicated DNA damage, while blue showed nuclei stained with Hoechst 33342; scale = 20 *μ*m. (g, h) Representative western blotting results showing the protein expression levels of SIRT3, p-P53-ser15, P53, OCT4, SOX2, or P21 in different groups. Protein expression levels detected by western blotting were quantified using ImageJ software and normalized to the control group. Approximately 10^5^ cells per well were seeded in 6-well plates. All quantitative data were obtained from three independent experiments and presented as mean ± SEM; ^∗^*p* < 0.05 and ^∗∗^*p* < 0.01 indicate a significant difference between the groups.

In addition to replicative senescence, the other type of cell senescence in this study is hydrogen peroxide-induced premature senescence. The hUCMSCs at PDL6 exposed to 100 *μ*M H_2_O_2_ for 24 h exhibited a notable increase in the senescent population marked by SA-*β*-gal staining, from 10.6% ± 3.7% (control) to 72.8% ± 2.6% (*p* < 0.05). To a certain extent, cells precultivated with 10 *μ*M SPD for 24 h before H_2_O_2_ treatment reduced the senescent population to 51.8% ± 5.9% (*p* < 0.05; Figures 4(a) and 4(b)). Similarly, intracellular reactive oxygen species (ROS) were significantly higher than in the control group. However, early SPD intervention significantly decreased intracellular ROS levels (Figures 4(c)–4(e)). Subsequently, western blotting was performed to detect the levels of senescence-associated proteins (p-P53-ser15, P53, and P21), stemness-associated markers (OCT4 and SOX2), and the longevity-related factor (SIRT3). Accordingly, the results showed that early SPD supplementation partially restored expression levels of these related proteins (Figures 4(g) and 4(h)). Additionally, DNA damage was detected by visualizing *γ*-H2AX immunofluorescence, which revealed that early SPD supplement decreased the H_2_O_2_-induced DNA damage in hUCMSCs (Figure 4(f)). The above results indicated that early SPD intervention protected hUCMSCs from H_2_O_2_-induced premature senescence.

### 3.5. SIRT3 Is Required for SPD to Exert Antisenescence Effects on hUCMSCs

**Figure 5:**
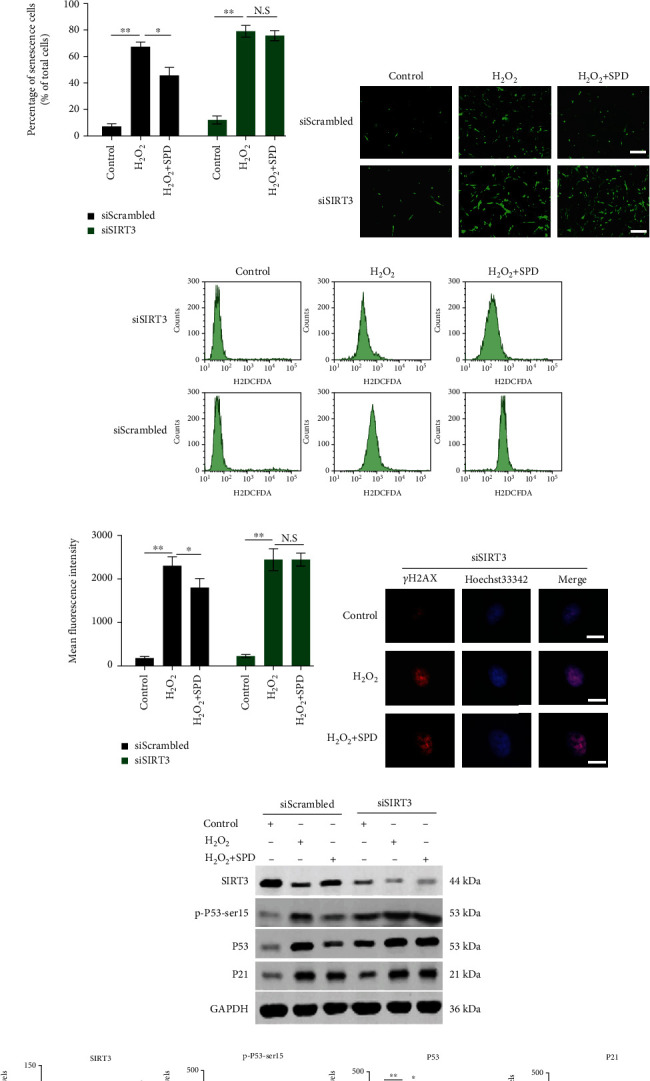
Silencing of SIRT3 offset the antisenescence effects of SPD on hUCMSCs. (a) The silencing efficiency of siRNA against SIRT3 (siSIRT3) or scrambled control (siScrambled) was assessed by western blotting and relative quantitative analysis. (b) A representative image showing SA-*β*-gal staining in siScrambled or siSIRT3 transfected hUCMSCs after SPD and H_2_O_2_ treatment, scale = 100 *μ*m. (c) The percentage of senescent cells stained in blue in each group was determined by quantitative analysis. (d) A representative image showing intracellular ROS levels by molecular probe H2DCFDA staining in hUCMSCs with indicated treatment; scale = 100 *μ*m. (e, f) Quantification of intracellular ROS levels was performed by fluorescence-activated cell sorting (FACS) analysis of stained cells to obtain the mean fluorescence intensity. (g) Immunofluorescence staining of *γ*-H2AX foci appearing red indicated DNA damage, while blue showed nuclei stained with Hoechst 33342; scale = 20 *μ*m. (h) Representative western blotting results showing the protein expression levels of SIRT3, p-P53-ser15, P53, or P21 in different groups. (i) Expression levels of SIRT3, p-P53-ser15, P53, and P21 were quantified using ImageJ software and normalized to the control group. Approximately 10^5^ cells per well were seeded in 6-well plates. All quantitative data were obtained from three independent experiments and presented as mean ± SEM; ^∗^*p* < 0.05 and ^∗∗^*p* < 0.01 signify a significant difference between the indicated groups; N.S.: not significant.

SIRT3, a major mitochondrial deacetylase that decreases mitochondrial ROS, has been shown to slow down senescence in multiple cell types. To evaluate whether SIRT3 has a role in the antisenescence effect of SPD, the SIRT3 gene was effectively silenced by siRNA against SIRT3 (siSIRT3; Figure 5(a)). Subsequently, the antisenescence effects of SPD on hUCMSCs were evaluated by SA-*β*-gal staining and intracellular ROS levels. Interestingly, a much higher percentage of the senescent population and ROS levels were detected after the SIRT3 gene was silenced (Figures 5(b)–5(f)). This suggested that early SPD intervention did not have a positive effect on the rescue of premature senescence induced by H_2_O_2_ when the SIRT3 gene was silenced. Furthermore, western blotting was performed to evaluate the expression levels of senescence-associated genes. The results showed that the changing trends of p-P53-ser15, P53, and P21 were highly consistent but negatively correlated with the tendency of SIRT3 (Figures 5(h) and 5(i)). However, when the SIRT3 gene was silenced, the expression levels of the senescence-associated genes increased substantially. Moreover, SPD intervention also reduced the inhibitory effect on the expression of p-P53-ser15, P53, and P21 (Figures 5(h) and 5(i)). Additionally, increased *γ*-H2AX expression indicated that DNA damage was also observed after SIRT3 gene silencing, suggesting that early SPD intervention did not alleviate the effect (Figure 5(g)). Therefore, the results suggested that SIRT3 was required for SPD to exercise antisenescence effects on hUCMSCs.

### 3.6. A Long-Term High-SPD Diet Delays Stem Cell Senescence *In Vivo*

**Figure 6:**
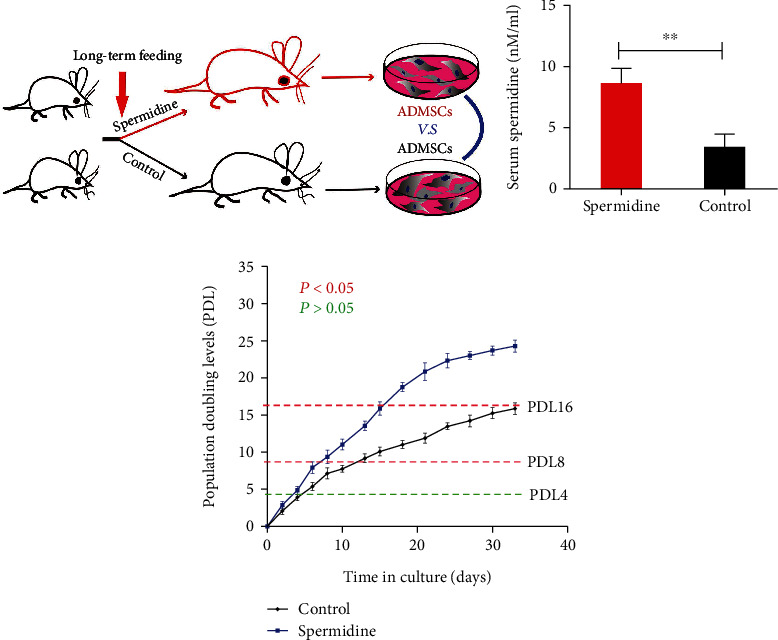
The effect of long-term spermidine administration to rats on the PDLs of rADMSCs. (a) Summary diagram for the experimental procedure *in vivo*. (b) The levels of serum spermidine in rats were determined after a 12-month spermidine feeding program. (c) After one year of feeding spermidine to rats, the replicative potential of rADMSCs was evaluated by the PDLs *in vitro*. The rADMSCs were derived from eight individuals randomly selected from 40 rats. The quantitative data were presented as mean ± SEM, and each group represented at least eight individual animals; ^∗∗^*p* < 0.01 indicates a significant difference between the groups.

In this experiment, to determine whether SPD delayed stem cell senescence *in vivo*, we applied a long-term high-SPD diet as a treatment factor (Figure 6(a)). The levels of serum SPD were determined after 12 months of feeding with SPD in rats. As expected, the plasma SPD concentration in the experimental group was significantly higher than in the control group (Figure 6(b)). Thus, a long-term SPD diet significantly increased the concentration of plasma SPD. Subsequently, to evaluate the replicative potential of rADMSCs after one year of SPD feeding, rADMSCs were isolated and expanded in culture. Notably, the primary rADMSCs from the experimental group required 15 days to reach PDL16, while the control group reached the same PDL in 30 days (Figure 6(c)). This suggests that long-term SPD feeding could maintain rADMSC levels *in vivo* with a high proliferation potential in aged rats.

**Figure 7:**
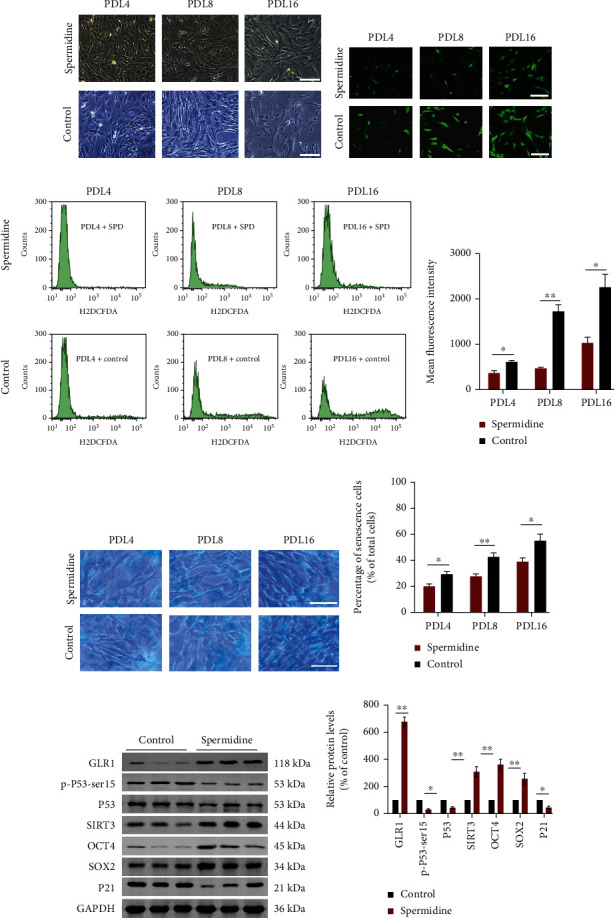
The long-term high-spermidine diet hindered rADMSC senescence *in vivo.* (a) A representative micrograph showing morphological changes of primary rADMSCs from different groups at the indicated time points *in vitro* after a one-year high-spermidine diet; scale = 100 *μ*m. (b) A representative image showing intracellular ROS levels by molecular probe H2DCFDA staining in hUCMSCs with indicated treatment; scale = 100 *μ*m. (c, d) Quantification of intracellular ROS levels was performed by fluorescence-activated cell sorting (FACS) analysis of stained cells to obtain the mean fluorescence intensity. (e) A representative image showing SA-*β*-gal staining in rADMSCs at PDL4, PDL8, and PDL16 with indicated groups, respectively, scale = 100 *μ*m. (f) The percentage of senescent cells stained in blue in each group was assessed by quantitative analysis. (g) Representative western blotting results showing the protein expression levels of GLR1, SIRT3, OCR4, SOX2, p-P53-ser15, P53, or P21 in different groups. (h) Expression levels of the corresponding proteins were quantified using ImageJ software and normalized to the control group. Approximately 10^5^ cells per well were seeded in 6-well plates. All quantitative data were obtained from three independent experiments and presented as mean ± SEM; ^∗^*p* < 0.05 and ^∗∗^*p* < 0.01 represent a significant difference between the indicated groups.

Observations of the cell morphology revealed that the rADMSCs from the experimental group kept a more regular shape during the long-term expansion *in vitro* compared with the control group (Figure 7(a)). Moreover, through intracellular reactive oxygen species (ROS) detection and SA-*β*-gal staining, we discovered that the intracellular ROS levels and senescent population marked in the experimental group were significantly lower than in the control group (Figures 7(b)–7(f)). Furthermore, western blotting was performed to evaluate the expression changes of related factors in stemness, aging, and antioxidation. The results showed that in contrast to the control group, the rADMSCs from the long-term SPD feeding group, antiaging factors, SIRT3, and GLR1 (glutathione reductase 1) were significantly upregulated, while the senescence-associated proteins, P21, p-P53-SER15, and P53 were downregulated (Figures 7(g) and 7(h)). This indicates that SIRT3 played a key role in the antiaging process of spermidine on stem cells *in vivo*.

## 4. Discussion

Long-term subculture leads to cell replicative senescence, which is characterized by the progressive loss of proliferation potential, increased SA-*β*-gal positive staining, increased ROS levels, DNA damage, and so on [40–42]. Spermidine, a naturally occurring small molecule compound belonging to the polyamine family, mainly exists in the semen. It also exists in common foods and is essential for the proper function of numerous metabolic processes [24].

In this study, for the first time, we demonstrated that early SPD supplementation resulted in a higher proliferation rate, decreased SA-*β*-gal activity, and lower protein levels of p-P53-ser15, P53, and P21 in later-passage hUCMSCs (PDL26). As a marker related to cell proliferation, Ki67 indicates the proliferation efficiency of cells. Thus, we detected that SPD intervention increased the expression levels of Ki67, indicating that SPD contributed to the proliferation of cells. Additionally, SIRT3, a major mitochondrial deacetylase localized to the mitochondrial matrix, reduces mitochondrial ROS and has been shown to slow down senescence in multiple cell types [33, 43, 44]. Interestingly, we observed higher SIRT3 protein levels in the PDL26-hUCMSCs with SPD intervention than those without SPD intervention. However, the protein levels of p-P53-ser15, P53, and P21 significantly increased when the SIRT3 gene was silenced, indicating that SPD lost its senescence-inhibiting effect. This suggests that spermidine delayed the replicative senescence of hUCMSCs by regulating SIRT3 involving p-P53-ser15, P53, and P21 [45–50].

The stemness-associated markers mainly include OCT4 and SOX2 [51, 52]. In this study, their expression levels decreased significantly during expanded culture but partially recovered after early SPD intervention. Additionally, FABP4, PPAR*γ*, ALP, and RUNX2 factors were considered indicators of adipogenic or osteogenic differentiation [53–55]. Moreover, the differentiation ability in the PDL26-hUCMSCs was lower than in the PDL6-hUCMSCs. Interestingly, early SPD intervention significantly facilitated the maintenance of adipogenic and osteogenic differentiation. These results suggested that early SPD supplement significantly delayed hUCMSC replicative senescence.

It is commonly recognized that reactive oxygen species (ROS) are the main factors that lead to premature cell senescence [40, 56–58]. In this study, early SPD supplementation decreased senescent populations marked by SA-*β*-gal staining and intracellular ROS levels. We demonstrated that early SPD intervention also inhibited H_2_O_2_-induced premature senescence. Furthermore, similar to cell replicative senescence, H_2_O_2_ treatment reduced the expression levels of SIRT3, OCT4, and SOX2 but increased the levels of p-P53-ser15, P53, and P21. Notably, early SPD intervention slowed down the process significantly. Moreover, oxidative stress induces DNA damage and telomere dysfunction is an important mechanism of cell senescence [59–64]. Herein, visualized *γ*-H2AX immunofluorescence staining was performed to detect the DNA damage. Results indicated that early SPD intervention remarkably decreased H_2_O_2_-induced DNA damage in hUCMSCs. A possible mechanism is that SPD protected DNA from adverse effects through the antioxidation effect or autophagy pathway. However, further studies are required to verify this hypothesis.

Throughout the whole study, we observed that cell senescence, including replicative and premature senescence, was positively correlated to P53 and P21, while it was negatively associated with SIRT3. SIRT3 is a member of the SIRT family and a major protein deacetylase localized to the mitochondrial matrix. It is a key regulator of mitochondrial function, which reduces both mitochondrial ROS and oxidative stress [65–69]. Given this, we speculated that the SIRT3 protein played a pivotal role in the SPD-mediated antisenescence process. Therefore, to test our hypothesis, the SIRT3 gene was silenced in hUCMSCs using three different siRNAs against SIRT3. As expected, SIRT3 knockdown led to a significant increase in the percentage of the senescent population, intracellular ROS levels, and DNA damage (*γ*-H2AX) when treated with H_2_O_2_. In contrast, early SPD intervention did not reduce these adverse effects. This suggested that SIRT3 silencing caused the loss of SPD-mediated antisenescence. Furthermore, western blotting was performed to evaluate the expression levels of SIRT3, p-P53-ser15, P53, and P21 [70–72]. The final results showed that the trends of senescence-associated proteins p-P53-ser15, P53, and P21 were negatively correlated with the trend of SIRT3 when the SIRT3 gene was not silenced. However, the expression levels of p-P53-ser15, P53, and P21 significantly increased when treated with H_2_O_2_ after SIRT3 silencing, regardless of SPD intervention or not. These results indicated that SIRT3 was required for the antisenescence effects of SPD on hUCMSCs. However, due to complex factors, determining the underlying mechanism requires further study.

To explore whether SPD delays the senescence of endogenous MSCs *in vivo,* a year of daily supplementation with high-dose SPD was performed in middle-aged SD rats in the experimental group, while an equal dose of drinking water was provided to rats in the control group. As expected, the long-term SPD diet significantly increased the concentration of plasma SPD. Next, primary rADMSCs were isolated and expanded in culture for one year. Interestingly, the primary rADMSCs from the experimental group showed a higher proliferation potential than the control group. Additionally, in contrast with the control group, the experimental group maintained a normal appearance for a longer time during *in vitro* expansion, while senescent populations marked by SA-*β*-gal staining decreased and intracellular ROS levels fell substantially. Based on previous studies, antioxidative factors, SIRT3, and GLR1 all play a vital role in delaying senescence [32, 73–75]. Furthermore, quantitative analysis of related factors indicated that SIRT3 and GLR1 were significantly upregulated, while senescence-associated proteins P21 and P53 [70–72] were downregulated. These data indicated that the antiaging effect *in vivo* of SPD progressed mostly by resisting oxidative stress. However, further studies are needed to clarify the underlying mechanism.

In summary, this study demonstrated that early SPD intervention significantly delayed the replicative senescence of hUCMSCs and inhibited H_2_O_2_-induced premature senescence. Also, SIRT3 was required for SPD-mediated antisenescence effects. Besides, this study also showed that SPD *in vivo* protected MSCs against oxidative stress and delayed cell senescence. Therefore, the results of this study verified that MSCs possessed the ability to proliferate and differentiate efficiently *in vitro* or *in vivo*, which is conducive to the clinical utilization of MSCs in the future.

## Data Availability

The data that support the findings of this study are available from the corresponding author on request.

## Abbreviations

MSCs: Multipotent mesenchymal stromal cells
SPD: Spermidine
hUCMSCs: Human umbilical cord mesenchymal stem cells
ROS: Reactive oxygen species
rADMSCs: Rat adipose-derived mesenchymal stem cells
GLR1: Glutathione reductase
PDL: Population doublings level
MFI: Mean fluorescence intensity
SVF: Stromal vascular fraction.
